# Book Reviews

**Published:** 1829-06

**Authors:** 


### CRITICAL ANALYSES.

Quæ laudanda forent, et quæ culpanda, vicissim

Illa, prius, cretâ; mox hæc, carbone, *notamus.*—Persius.

*An Essay on the Use of the Nitrate of Silver, in the Cure of Inflammations, Wounds, and Ulcers.* By John Higginbottom, Nottingham, Surgeon. *Second Edition, much enlarged and improved.*—8vo. pp. 204. Seeley and Burnside, London, 1829.

Having already noticed the first edition of Mr. Higginbottom's work, we did not, at first, think it would be necessary for us to do more than announce the receipt of the copy of the edition now before us. But, in examining it more fully, we are struck with the many and important additions which the author has continued to make to our knowledge of the use of the nitrate of silver. The external application of this substance is not only useful in the healing of various ulcers, but, what we did not previously imagine, in cases of external inflammation, of erysipelas, of bruised as well as punctured wounds, and of burns.

Mr. Higginbottom appears to have fairly established the utility of the nitrate of silver as a *new kind* of remedy in many surgical diseases. It appears directly to subdue the actions of the phlegmonous and the erysipelatous inflammation; and to change that of the suppurative, the sloughing, and the ulcerative, to the adhesive. The nitrate of silver, applied along the course of inflamed absorbents, directly subdues their morbid action, all tenderness promptly subsiding; applied lightly to burns, the inflammatory action characteristic of this accident is promptly exchanged for one of a milder and less painful kind. We perfectly agree with the author, both from what we ourselves know and from the facts here laid before us, that the nitrate of silver has very improperly been denominated a caustic. Instead of destroying, as Mr. Higginbottom justly observes, it more frequently preserves parts which would inevitably slough but for the preservative powers of this remedy. We know no fact which better illustrates this property of the nitrate of silver, than that of its preventing the formation of pits from small-pox; an effect obviously depending upon the sloughing of minute portions of the subcutaneous cellular membrane. It is suggested in an early part of this volume, and the remark is repeated from the former edition, that the nitrate of silver may be useful in some cases of compound fracture, by affording an effectual means of closing the external wound, and excluding the atmospheric air. This object is certainly most highly important, and we think Mr. H.'s suggestion far more likely to succeed than that of Sir Astley Cooper, of applying lint dipped in blood: the eschar is more tenaciously adherent; and the application of the nitrate of silver may induce a better, because a more adhesive, state of inflammation of the bruised parts, and even of the ends of the fractured bone.

But to proceed to a more regular analysis of this useful little work.

Some cases are given illustrative of the effect of the nitrate of silver in subduing the inflammation of phlegmon, or a line of inflamed absorbents; in arresting the spreading of erysipelas; and in preventing and modifying the formation of pus.

"It is frequently only necessary to convert the cuticle into an eschar over the inflamed surface. In other cases, the nitrate of silver must be applied more abundantly, so as to induce a degree of vesication. The part is first to be washed with soap and water, to remove any oily substance from the skin, and then it is to be wiped dry; the inflamed and surrounding skin is then to be moistened, and a long stick of nitrate of silver is to be passed over the moistened surface, taking care that not only every part of the inflamed skin be touched, but the surrounding healthy skin, to the extent of an inch or more beyond it, in severe cases. The nitrate of silver may be then passed over these surfaces once, twice, thrice, or more times, according to the degree of inflammation: once in slight cases, twice or thrice in common cases, and more frequently if quick vesication be required. It is necessary to apply the nitrate of silver more freely on the hand or sole of the foot, where the cuticle is thick, than on other parts. After the application, the part is to be exposed to the air to dry, and is to be kept cool."

Mr. Higginbottom observes, "that whenever an eschar made over a wound or ulcer can be preserved adherent, such wound or ulcer infallibly heals;" and he goes on to describe shortly the immediate effects of the nitrate of silver applied to such parts. In order to form this adherent eschar, several things should be particularly attended to: first, the application of the nitrate of silver should be made not only over the whole surface of the wound, but also upon the surrounding skin. 2d. It should be applied lightly over the wound, so as to touch every part of it; and, if the surrounding skin be inflamed, it should be moistened with a little water, and the nitrate of silver passed once lightly over it. 3d. Every thing must be avoided which might detach it, but, as it separates from the healed edges of the wound, it should be carefully removed by a pair of scissors. To preserve the eschar adherent still more effectually, it is advised that a small portion of gold-beaters' skin be applied over it, which can be removed by wetting it with a little water, when it becomes necessary to let out any matter which may have formed under the eschar. Mr. H. here draws a comparison between the mode of healing by eschar and that by scabbing, and plainly shows that the former is to be preferred.

We will begin our observations with phlegmonous inflammation; and here let it be observed, that Mr. Higginbottom generally prescribes an emetic and purgative, to clear the primæ viæ. Miss ——, aged twenty-one, of a gross habit, was seized with an acute pain (on Saturday) across the patella, whilst at dinner, which continued getting worse. In a few hours more, inflammation was observed spreading up the thigh, when the poultice was taken off, and soap liniment used. The poultice was again applied. When Mr. H. saw her (on the Monday,) "the inflammation had spread along a third part of the length of the thigh, and downwards nearly to the ankle. The limb was much swollen, exceedingly hot, and a slight fluctuation was felt just below the patella. I applied the nitrate of silver over the whole surface of the inflamed parts, and did not open the abscess, as I knew from experience that the tumor would subside, rather than increase, soon after the application of this remedy. I directed an emetic and purgative medicine, and desired a fracture cradle to be put over the limb, which was kept exposed. On Tuesday, the inflammation was quite arrested, and there was no heat. I opened the abscess below the skin, and some fluid was evacuated, which had more a serous than a purulent appearance. I applied the nitrate of silver within the cavity of the abscess." On the following day there was an increased inflammation below the part on the foot, attended with swelling. The Argent. Nit was again applied; and on the following Wednesday the patient was convalescent, and only required a bandage till the swelling was gone.

Four cases of this kind are related.

In whitlow, when suppuration has commenced, Mr. H. advises the part to be opened freely, and the Argent. Nit. to be applied within the opening, and then the part to be enveloped in a cold poultice. In slight cases, the Argent. Nit. is advised to be passed slightly over the part, and in this manner suppuration and a continuance of the inflammation has been prevented.

Mr. H. observes, that he does not at first use the nitrate of silver in erysipelas, but resorts to every active constitutional means of cure. Six very interesting cases of this kind are given; two are related in a letter by Dr. Storer, addressed to the author. We transcribe Dr. Storer's letter, as he was an impartial observer of the effects of the remedy.

"Your late treatise had apprised me of your mode of using lunar caustic in the treatment of certain wounds and ulcers. I had not lost sight of the facts there detailed, and have been gratified to find, by repeated inquiries, that this plan of treatment continues to be confirmed by its success in your hands, and by other surgeons who have adopted it.

"It is now understood that it is capable of being extended with signal benefit to old and extensively ulcerated legs, of which one case that I have seen is certainly an example. In cases of local disease, such success does not exceed the ordinary bounds of expectation; nor is it without an analogy to the general application of this substance. When I heard that for some time you had adopted the same plan of extinguishing cutaneous inflammation, not merely in symptomatic erysipelatous affections, but also in that constitutional erysipelas which is often epidemic, and always ushered in and accompanied with fever, I confess that I could expect no success from a practice militating so directly against the views generally entertained of the nature of this disease, and would give no ear to it without ocular demonstration of its utility. I have seen two cases of constitutional erysipelas treated upon this principle, and, as far as two cases will go to authorize a conclusion, mine must be greatly in favor of the practice. The first was that of a healthy young woman, upwards of twenty years of age, seized with rigor, followed by smart fever, and on the second day by a complete erysipelas of the face, extending to the hair on the forehead. The fever continuing, with a further extension of the inflammation, after bleeding, purgative and saline medicines, and a small vesication having arisen on the cheek, it was judged necessary on the third day to apply the caustic over the whole extent of the erysipelas. The patient's own account was, that she suffered considerable pain for ten hours, but from that time the feverish symptoms ceased, and the inflammation was arrested and subdued. When I saw her, the fourth day after the application of the caustic, there was neither fever nor any remains of erysipelas. The face was as black as an African's; but, in a few days more, I found her free from all symptoms of disorder, the epidermis peeling off, and the complexion underneath quite natural.—The other was a fine healthy boy, sixteen months old, who, after a feverish attack, had erysipelas on one hip and thigh, which extended partially to the leg. In this case also the fever and inflammation had subsided in forty-eight hours and upwards after the application of the caustic. The account of the child's mother was, that it cried very much for an hour after, then fell into a long and calm sleep, out of which it waked without fever, and calling out for food. In three days after, when I saw the child, it was quite well, and the scarfskin of the inflamed parts separating. This is the extent of what I have observed of this novel mode of treating erysipelas, and which has surprised as it has satisfied me. Sincerely wishing success to your endeavours to improve the practical part of your profession, I am, &c.

"John Storer."

Out of five cases as relating to the effect of this remedy in inflammation of the absorbents, there are two briefly related by Dr. M. Hall, in a letter addressed to the author. One of these we will give in Dr. Hall's own words:

"The case was that of a young lady, aged twelve years: it began by a chilblain upon the heel; inflammation of the absorbents up to the groin followed; suppuration took place in twenty-four spots. The nitrate of silver was not applied during the first five weeks: this was very much regretted after its effects had been observed. The patient was seen by Mr. Lawrence and Mr. Wardrop, who both expressed themselves much interested in it. As the case would be long, I shall simply enumerate the effects of the application of the nitrate of silver, as they presented themselves to my observation:

"1st. It prevented suppuration in many places where the redness and tenderness were recent, yet such as before had inevitably led to the formation of pus.

"2d. If suppuration had taken place, the tenderness was still promptly removed, and the pus, from being thick, white, and opaque, was rendered first thin and somewhat limpid, and perhaps streaked with blood, and then by degrees perfectly watery and limpid.

"3d. Where the pus approached the surface, a small opening formed, by which it exuded, and it became unnecessary to use the lancet.

"4th. The abscess was far more disposed to heal than any of those to which the nitrate of silver had not been applied."

Dr. Hall observes, that he has seen the early application of the nitrate of silver along the course of absorbents subdue the disease at once.

Mr. Higginbottom's mode of treating punctured wounds will be best explained by giving two or three cases:

"I.—A servant maid, aged twenty-four, applied to me with a swelling of the middle finger, and of the back part and palm of the hand, attended by such pain as to prevent her sleeping in the night. She thought this affection had arisen from a puncture by a pin or needle in washing. On examination, I perceived a small wound at the middle of the finger, at the first joint; and, on removing the skin with a lancet, a little pus escaped, and left a very small cavity. I applied the nitrate of silver within this cavity and over and beyond the inflamed parts of the finger and hand, previously moistened by water, and I left them exposed to dry. I prescribed an emetic and purgative medicine, and desired that the hand might be supported in a sling. On the following day, my patient stated that her hand was perfectly easy, and had been free from pain from the time the sense of heat, occasioned by the application, had subsided; she had passed a good night; the inflammation of the hand was completely checked in its progress; the swelling remained as before. On the next day, the patient made no complaint; the swelling had become soft and puffed to the touch. In a few days more, the cuticle began to peel off, and in one point, where it was thick, there was a slight degree of tenderness. From this time there was no further trouble or complaint.

"II.—Mr. Cocking's son, aged twelve, received a stab in the palm of the hand from a penknife, which has been followed by much swelling and pain, the punctured orifice being nearly closed. I applied the nitrate of silver within the puncture, and directed a cold poultice to be laid over the whole hand. On the following day, I found the poultice had not been applied; there were more pain and swelling; an eschar was formed over the puncture, which I removed, and thus gave issue to a considerable quantity of pus. I again enjoined the application of a cold poultice, kept constantly moist with cold water. On the succeeding day, the inflammation had greatly subsided. I repeated the application of the nitrate of silver, and poultice. On the fourth day, the inflammation had nearly disappeared, and on the fifth, entirely.

"In such cases, the nitrate of silver unites the advantages of at once opening the puncture and of subduing the inflammation, thus preventing the formation of deep-seated abscesses."

**III.—Mr. Higginbottom gives the following case in order to point out the mode of treatment he would recommend in wounds received in dissection; and this seems to coincide entirely with that Sir A. Cooper advises.**

"About eight o'clock in the morning, I received a puncture in the examination of a puerperal case: I merely washed the part. About four o'clock, my attention was called to a sharp pain in the punctured part, on examining which there appeared a little elevation of the skin. In three or four hours more, there was more swelling round the puncture; and at this point there was a small tumor, about half the size of a large shot-corn. I experienced a little headach, to which I was quite unaccustomed. I applied a spirituous lotion, and went to bed. In the night I had an increase of pain, and became feverish and restless, with increase of headach; the thumb and hand were becoming much more swelled, and the absorbents up the arm inflamed. I took an emetic, and afterwards a dose of calomel, followed by a saline purgative and an enema; and I applied the lotion constantly to the hand and arm. Afterwards I began to take two grains of the Submurias Hydrargyri every three or four hours.

"About four o'clock on the following day, I experienced excessive pain in the punctured part: I therefore particularly requested that I might have a free incision made into it, though I knew no suppuration could have taken place. This was done by the lancet. The incision gave considerable relief to the part, and for a short time the pain of the head was better. A large cataplasm was applied. In the course of the evening I experienced a slight chilliness, with violent fits of vomiting, which continued for about half an hour; this was succeeded by great heat, and afterwards by profuse perspiration, at which time I became much easier, until it was followed, in the course of about half an hour, with another fit of chilliness and vomiting; which was again succeeded by a hot fit and perspiration; which were repeated at intervals for twelve hours, the second being attended with very violent heating pain in the head. The hand was now become twice its natural size, and the arm was considerably swelled as high as the elbow, with some enlargement of the axillary glands. The tonsil on that side of the throat was also a little enlarged and painful. My medical attendant recommended the loss of blood: twenty ounces were therefore immediately abstracted, with decided relief of every symptom.

"The calomel had begun to affect the gum; the saline purgative was continued. I had no return of the vomiting, but was in every respect better.

"It is my opinion, from what I have since experienced, that this severe and painful illness would have been entirely prevented by the free application of the nitrate of silver, and that it would have been the only local remedy necessary. In the second state of the puncture, where a small tumor is raised, I would remove this tumor by the lancet, before applying the nitrate of silver to form an adherent eschar, and I would adopt the precaution of applying it on the surrounding skin, and beyond the swelling. At a later period still, I am of opinion that, when the hand had become much swollen and tense, and the inflammation had spread up the arm, the external application of the nitrate of silver would have subdued and checked the inflammation, and that the constitutional affection would have ceased."

This remedy is recommended to inflamed leechbites, and to the bites of animals.

Here we will give a short passage in the author's own words, as relating to this application to bruises, in which he strongly recommends it, and gives some cases illustrative of its good effect.

"In this place I have, indeed, to make an observation of particular interest, both in a pathological and curative point of view: it is that the formation of the slough has always been prevented by an early application of the nitrate of silver, in the cases which have hitherto fallen under my care. This fact may probably admit of explanation in the following manner: the bruise partially destroys the organization of the part, and the subsequent inflammation completing what the injury had thus partially effected, a loss of vitality takes place, and the slough is formed. The early application of the nitrate of silver has already been shown to have the remarkable effect of preventing the inflammation consequent upon certain wounds; and in this manner, in the case under consideration, the part recovers from the injury done to its organization, and its vitality is preserved, one of the causes of the slough being removed.

"CASE.—J. Jennings, a bricklayer, aged twenty-six, fell through the roof of a house, and bruised and lacerated his shin rather severely, to the extent of an inch and a half in one part, and in a less degree in several others. I applied the nitrate of silver to the wounds immediately. On the following day the eschar was found to be adherent, and there was neither pain nor swelling. The eschars separated in nine days, leaving the wounds healed. It is remarkable that the eschar remains a greater or less time over the wound according to the severity and exigency of the case. This case being less severe than the former, one of the eschars remained upon the wound during a much shorter period of time.

"Mrs. Willoughby, aged fifty-one, received a blow on the arm, which caused a wound, and broke the radius: the wound, however, did not communicate with the broken bone. The part was inflamed, but the arm itself was not swollen. I saw her immediately after the accident, and applied the nitrate of silver on the whole of the forearm, and over the wound. I put the arm upon a splint and sling, and prescribed a dose of the Submurias Hydrargyri and a dose of purgative medicine. On the following day she complained of little or no pain, and had very little smarting from the application of the nitrate of silver; and there was less swelling than usual in such cases. The eschar was adherent over the wound; there was slight vesication on the arm. The next day she expressed herself as surprised that she had had so little heat or pain. Two or three days afterwards the eschar was adherent all over the arm, and nothing was necessary but to keep it in the splint till the bone should be united."

Mr. H. has not yet had an opportunity of adopting this mode of treatment where the larger bones have been broken, but he feels very little doubt that the trial will be successful. No evaporating lotion, or other means which he has ever yet seen employed, has the same power of preventing and subduing external inflammation in such cases as the nitrate of silver.

Mr. H. has abandoned healing ulcers by eschar, excepting in those cases where the ulcers are small and free from inflammation, where there is little discharge, and where the parts are not exposed to much motion or friction.

"The plan of treatment which I have more recently adopted in large ulcers attended with inflammation, is far more successful, and requires very little care or attention on the part either of the surgeon or patient. If there be swelling or œdema, I direct the patient to take a dose of opening medicine, to apply a common poultice of bread-and-water over the ulcer, and to keep in bed for four-and-twenty hours. The inflamed parts must be washed with soap and water, and wiped dry. They are then to be moistened with water, and a long stick of the nitrate of silver must be passed all over the inflamed and ulcerated surfaces, twice, and rather more freely on the ulcer itself and on the surrounding skin. Lint must then be put on the ulcer, and the whole of the inflamed and ulcerated parts must be covered with the neutral ointment spread on linen; a compress, with five or six folds of fine linen, is then to be applied over the ulcer, and a common roller, not too tight, to keep on the whole. The leg is to be examined on the fourth day, when it will be found that the inflammation is nearly, if not entirely gone, and the ulcer in a healing state. The nitrate of silver must then be applied on the whole of the ulcer, and once lightly over the skin immediately surrounding it, one or two inches in breadth; the lint and ointment are to be applied as before, and the bandage applied rather tighter. The case must be treated in this manner every third or fourth day until the ulcer is healed. I would recommend wearing a calico roller for some time afterwards, till the leg has recovered its usual strength. The patient may walk about after the first or second application of the nitrate of silver."

The foregoing observations, as relating to the treatment of ulcers, are of the greatest practical utility; and every surgeon who has had much experience among the poor, either in private practice or in attendance on a dispensary, we are assured will agree with us; for there are no cases (treated as old ulcers of the legs have been hitherto,) which so much exhaust the funds of a dispensary or take up so much of the surgeon's time, and, we may add, which produce such protracted suffering to the patient.

The cases of small ulcerations we shall pass over, and give some of the cases of ulceration with inflammation, and those of old ulcers, as in at least ten or twelve cases of the latter we have seen the remedy attended with the most marked success, and in all the usual mode of treatment had been adopted and persevered in for some months, without the least benefit. Mr. H. adds occasionally to his usual mode of treatment the plan recommended by Mr. Baynton and improved by Mr. Scott.

I.—Mary Williamson, aged sixty-nine, fell down and broke her shin: this was followed by swelling. She rubbed the part with the soap liniment, which caused inflammation. She afterwards applied a poultice, but the leg still got worse for six weeks. She then went to an infirmary, and remained under surgical care for some time. The sore was now healed, but the inflammation still remained over the whole of the leg. The patient then placed herself under the treatment of an empiric for three weeks. Still uncured, she returned home into the country, and attempted to work at a cotton factory. This she did with much suffering, and soon became quite unable for any avocation.

"She came to Nottingham, and fell under my care. At this time the whole of the leg was inflamed, and affected with œdema. A large abscess had formed over the malleolus internus, and a small one on the opposite side. There were several ulcers on the shin, from the size of a crownpiece to that of a sixpence. I prescribed a dose of opening medicine, and directed her to keep in bed for twenty-four hours. I washed the leg afterwards with soap and water, opened both abscesses with the lancet, and applied the nitrate of silver over the whole extent of the inflamed surface, over the ulcers, and within the abscesses. I then applied a poultice to each of the abscesses, lint upon the ulcers, and the neutral ointment over the whole of the inflamed parts, in the manner already described. In four days, the patient having still kept her bed, all inflammation was gone, and the ulcers and abscesses were in a healing state. I again applied the nitrate of silver to the ulcers and abscesses, and dressed them with lint and the neutral ointment. In four days more there was very great improvement; the abscesses were nearly well, and the ulcers healing rapidly. In other four days the abscesses were healed, the ulcers healing fast.

By four more applications, the ulcers were all made well. It was necessary in this case to continue the application of a bandage to the leg twice a week for several weeks, and to apply the nitrate of silver to parts which became slightly inflamed from time to time.

"II.—Mrs. A. had been subject to ulcers of the right leg for seventeen years; she had had regular surgical attendance for a year or two at a time; but, as is too customary in such cases, the ulcers had always broken out again. This case had not lately been attended with much inflammation. Mr. Baynton's plan having been used for two years, only one ulcer remained: this was situated on the outer ankle, and was about the size of a sixpence, and not deep; the surrounding skin was very hard, and she complained of an intolerable itching, for which cold water, applied through the medium of a bandage, gave but very temporary relief. She had for years had all the symptoms consequent upon such ulcers; she had suffered much from loss of rest, and was totally unable to follow her employment. I passed the nitrate of silver upon the ulcer, and over the surrounding inflamed skin, and I applied lint, and the neutral ointment spread on linen, over the ulcer; and I continued the bandaging according to Mr. Baynton's plan. In five days all inflammation and itching were gone, and the ulcer was healing. In three more dressings, on each third day, the ulcer quite healed.

"It is now two years since this case was cured, and the patient has had no return of inflammation, irritation, or ulcer. She has washed the leg with cold water every morning, and constantly applied calico bandages.

"III.—Timothy Coleman, aged thirty-two, whilst in a state of intoxication, burnt his shoulder and arm very extensively. He was under the care of a surgeon, and the sore was healed in ten weeks. There still, however, remained an inflamed surface, larger than the size of the hand, over the deltoid muscle. It had the appearance of a fungus cicatrised over; it was attended with so much heat and pain as to prevent him from sleeping at night, or following his employment in the day, for thirteen weeks, even after it was said to be cured. He had used a number of remedies. His health continued good.

"I first saw him on June the 20th, 1827. I applied the nitrate of silver, as in external inflammation, over the whole diseased surface. I directed the part to be exposed to the air for three days, then to be covered with the neutral ointment. My patient resided at a distance in the country. I did not see him again for a fortnight, when he informed me that, eight hours after the application of the nitrate of silver, he had more ease than he had experienced since the accident; that he was nearly free from pain, and slept well. I again applied the nitrate of silver very freely on the whole affected surface, as there still remained several inflamed spots, besides several slight ulcerations caused by the nitrate of silver. I

then covered the part with the neutral ointment. In another week I saw my patient again. He said he had suffered more from the last application than from the former one; that it had acted more like a blister, that there had been a very free discharge, and that the eschar had separated sooner. There appeared, however, scarcely any irritation, except from a few superficial ulcerations, on which I passed the nitrate of silver very lightly; I continued the neutral ointment. A few weeks afterwards this man called on me to say that he was quite well. This peculiar case is almost incurable by any other means."

There is a short appendix to the volume, giving some cases illustrative of the use of the nitrate of silver in disease of the knee-joint, inflammation of the urethra, gunshot wounds, neuralgia, ulceration of the tongue, and irritative ulceration near the eye, and fungous ulcer of the navel of infants. It also contains a letter from Mr. Webster, of Dulwich, to Dr. M. Hall; and another from Mr. J. Brown, Camberwell, in which the latter gentleman communicates two cases, one of a delicate female, who, after the birth of her first child, wasted in flesh, and was subject to leucorrhœal discharges, with great debility; her general health was subsequently in other respects much deranged, and at length her wrist-joint and thumb became enlarged and painful. She had consulted Sir A. Cooper and Mr. Abernethy, and, although her general health was somewhat relieved by their prescriptions, the enlargement of the wrist was no better; and, in addition to this, her elbow was now swelled and extremely painful. These local affections were cured by the nitrate of silver, and her general health was restored by the sulphate of quinine. The other case was that of a laceration of the fascia of the forearm, depriving the patient of the power of using the limb: this was also cured by the application of the nitrate of silver.

Mr. Webster has written a long letter, in which he has suggested a theory to explain the action of this remedy. He has employed it with benefit in a case of extensive burn, stricture, &c.; he has also applied it in some cases of a peculiar affection of the larynx, of which he gives a description. The three first cases proved fatal; in the third, the only remedy out of a great number which proved at all beneficial was the application of the nitrate of silver: this was used at the suggestion of Mr. Wardrop. Mr. Webster, addressing Dr. Marshall Hall, observes,

"It was the relief afforded in this case, and some conversation I had with you on the use of the nitrate of silver in erysipelas, &c. which induced me to give the remedy a full trial in the following one:

"Jane Thornback, aged four years, has been complaining for a fortnight of languor, debility, headach, and other feverish symptoms. About a week ago she first noticed that her throat was sore. She had difficulty in swallowing or articulating, and the tonsils became enlarged externally. Yesterday her respiration was affected, and there supervened a short, dry, ringing cough, with an inclination to vomit, and an occasional sense of strangling. The present symptoms are, considerable emaciation; pale, sunken, and anxious countenance; purplish tinge of the lips; respiration difficult and increased in frequency; the inspiration sonorous and rattling, the alæ nasi acting rather strongly; the throat is painful, especially in swallowing; the uvula and both tonsils are swelled and covered with sloughs, the margins ulcerated, the surrounding parts of a dark red colour; the pulse 120, and weak."

After describing the above case and its treatment, with the post-mortem examination, much more at length than the limits of a review will enable us to do, Mr. Webster observes: "Though this case proved fatal, yet I should, if another instance of this intractable complaint presented itself, pursue the same remedies; as, in three cases, two recovered, which, in my experience of the three previous ones, I think I might fairly say, under the ordinary treatment, would also have proved fatal."

Mr. Webster has made some remarks on the superior advantages of the nitrate of silver to cantharides for the purpose of blistering. In these observations we fully agree with him, and must add that we have seen more than one case where the local irritation produced by the Emplast. Lyttæ has been so excessive, and the sore thus caused become so unmanageable as to produce death: this, we feel convinced, would not have occurred had blistering in these cases been produced by the nitrate of silver.

We will observe, before taking leave of our author, that we have employed the remedy within the last few days, in a case of enlargement and stiffness of the first joint of the thumb, the consequence of a wound into the capsule: with three applications this inconvenience was removed.

We hope Mr. Higginbottom will continue his investigations respecting this singularly useful remedy. In the mean time we recommend our readers to peruse this little volume, and judge of its merits for themselves. It is purely practical, and therefore well calculated to interest and instruct the great body of the profession.

*Further Observations on the Use of the Lancetted Stilettes, in the Cure of permanent Strictures of the Urethra: with additional Cases.* By Richard Anthony Stafford, Member of the Royal College of Surgeons, and lately House Surgeon to St. Bartholomew's Hospital.—8vo. pp. 64. Longman and Co. London, 1829.

Some surgeons contend that there are no cases of stricture of the urethra which may not be overcome by the patient and repeated use either of the wax or metallic bougie. This opinion is, however, but very partially advocated, and it is generally admitted that instances not unfrequently occur in which none of the ordinary instruments can be passed into the bladder, whatever may be the manual adroitness or perseverance of the practitioner. Assuming, then, that we have to deal with a case of stricture which is totally impermeable, our alternations of treatment have hitherto been two: either to attempt the destruction of the stricture by the application of caustic bougies, or to cut down upon the urethra, divide the stricture with the knife, and then endeavour to restore the natural passage by the use of the catheter and the union of the external wound. The first of these modes of treatment was once thought to be almost infallible and free from danger. More extensive observation has since shown the contrary, and it is now well known that the caustic frequently fails, and that the use of it is often followed by profuse hemorrhage and other alarming symptoms. Even when it does succeed, the process of cure is usually very tedious. The operation of dividing the strictured part by incision is generally successful, if it is skilfully executed. That it is a painful one, and at all times formidable to the patient, must be evident, although we would not, with Mr. Stafford, draw any comparison between it and the operation for lithotomy.

The plan proposed by Mr. Stafford to avoid the uncertainty and hazard of the one mode of treatment, and the pain inflicted upon the patient by the other, we formerly described* in our notice of his first work upon this subject. The object of Mr. S., in the publication of these "Further Observations," is to prove, by additional evidence, that the apprehensions which have been entertained respecting the safety of his instruments are imaginary, and that the success of their employment is greater than that to be expected from any other mode of treatment. Mr. Stafford has ope-

* London Med. and Phys. Journal, September 1828, p. 240 et seq.

rated with the instruments he proposes more than twenty times, without the slightest dangerous symptom occurring at the time or afterwards. He has divided strictures in the urethra, in almost every part of its course, at distances of one, three, four, five, and six inches from the orifice, at the point immediately behind the bulb, and throughout the whole membranous portion. Some of these strictures were half an inch, others an inch, and one was two inches in length. He operated at one time on four strictures in the same urethra, varying from one fourth of an inch to above an inch in extent; and in no instance was there a symptom to occasion even slight alarm.

"The small quantity of blood lost from the operation was surprising, only in one case amounting to a tablespoonful, and usually not exceeding a few drops or a teaspoonful. This fact is so extraordinary, that, unless there had been repeated proofs, it would hardly be credited. The inflammation which has occurred has never been very great; and, when it has taken place, I am much inclined to attribute it to the irritation excited by the catheter having been left in the bladder. I am the more confirmed in this opinion from the fact that, in the only case in which I omitted its introduction, no sensible inflammation followed.

"The superiority of the division by the lancetted stilettes over the only plan of treatment which can be brought in competition with it, that by the caustic, is evident from the following circumstances: The pain is much less. This was admitted by every patient who had experienced both plans of treatment. In truth it is so little as, by their own confession, to be not worth mentioning. As a proof of this, all my patients stood during the operation, which did not usually occupy a longer time than a period varying from one to two minutes. The bleeding is not so great as what often attends the passage of a common bougie, consequently very much less than that after the application of caustic, in which the loss of half a pint or a pint of blood is no uncommon occurrence. The formation of a false passage, which, in the most experienced hands, will inevitably sometimes occur from the use of caustic bougies, has never resulted in any case where I have employed the instruments. The last, and perhaps principal, proof of superiority, however, of this plan of treatment, is the shortness of time occupied, and the rapidity of the cure. The length of time necessary for the common method, of course, varies indefinitely: three months may be stated a short period; and it often extends to one or two years, with a great chance of the recurrence of the disease in a more aggravated form. On the contrary, the longest time which it has been found necessary to pass a bougie after dividing the stricture with the lancetted stilette, has never exceeded six weeks; and in those cases it was passed merely to satisfy myself and the patient of the nonexistence of the disease. Usually a large-sized bougie has been introduced almost immediately after the operation, and the cases have not required attendance more than three weeks or a month." (P. 9.)

A perusal of the cases will show that the cures were complete.

Mr. S. has examined the urethra after an interval of one or two years, and he has been able to pass a large-sized catheter without difficulty, and the patients have made water in a natural manner. When he published his former work upon this subject, the author had had no opportunity of ascertaining the state of the urethra after death: he has now a preparation of the urethra.

"His stricture, which was one of twenty-three years' standing, and more than an inch in extent, was situated at the bulb, and in the membranous portion. The operation was completely successful, and he made water naturally. He lived nearly two years without any symptoms of stricture, and died, æt. seventy-six, from diseased lungs, and the infirmities of old age.* On examination, the caliber of the canal was found natural throughout; there was no hardness round the part that had been formerly contracted, and the membrane lining it was continuous with the rest of the urethra. The only difference that could be perceived was a little redness and roughness, and the incisions made by the instrument could be traced, though not very distinctly." (P. 12.)

The present pamphlet contains a relation of eleven cases, in addition to those formerly detailed. We shall give an abstract of each.

Case I.—F. J. Esq. applied to the author, at Brighton, on the 8th August, 1828. In the autumn of 1799, a difficulty in passing the urine was first experienced. Stricture was detected about three inches down the urethra, and the caustic bougie was employed at intervals for about a month. Considerable effusion of blood followed each application of the instrument. The patient was then discharged by his surgeon as cured. In 1820 the complaint returned, and Sir Everard Home discovered the stricture was formed in the same place as before. The caustic was now employed but a few times, as it caused great pain and irritation in the urethra, and very distressing rigors were brought on. A metallic bougie was passed every third or fourth day. In two months relief was obtained, but not a cure. Bougies were occasionally passed by the patient himself until 1826, when he became so much worse that the urine could only be

* "The name of this man was John Sych (whose case is related at page 143). On his coffin his age was marked seventy-six; so that he must have been more than seventy-four when operated on."

voided drop by drop. Pain in the urethra excessive, and, from straining, great soreness in the abdomen and loins. General health much affected. The smallest bougie could only be passed, and even that with much pain and difficulty. The caustic was again used several times, as the case continued obstinate: it failed in producing the desired effect, and gave rise to symptoms threatening retention of urine. It was therefore abandoned. The symptoms continued increasing in severity, and at length an attack of retention of urine occurred.

Mr. J. now placed himself under the care of Mr. Stafford. At this time the health and spirits of the patient were almost exhausted. He had not made water for twelve hours, and had only passed it by drops, and involuntarily, for two years. To relieve the excessive irritation of the urethra, which prevented the introduction of a bougie, twelve leeches were applied to the perineum; fomentations, warm bath, and opiate injections. A bougie, the size of a knitting needle, was now passed. The stricture was three inches from the orifice. The day after, Mr. S. operated with the double-lancetted stilette over the wire. The patient stood up during the operation, which lasted about one minute and a half; it caused but little pain. Only a few drops of blood followed the incisions. The stricture was divided throughout its length without withdrawing the instrument; the catheter could only be passed as far as the membranous portion: here the spasm was so violent that the author was obliged to desist. A No. 9 bougie was left in the urethra through the divided stricture. Leeches and fomentations were immediately applied. In the evening the patient withdrew the bougie, and voided the urine in a full stream, and with little pain. A week after the operation was performed the urethra appeared quite healed, and a catheter, No. 8, passed easily into the bladder. This was repeated twice a week for a month, gradually enlarging the size of the catheter to the natural caliber of the urethra. The health of the patient was rapidly restored.

Case II.—W. Chaters, æt. fifty, unhealthy and emaciated, admitted into the Marylebone Infirmary; has had a stricture for twenty-eight years. About eighteen years ago the caustic was applied several times during the space of three months, without benefit. He was considered incurable, and ever since has suffered the worst symptoms of stricture, and passed his urine *guttatim,* or involuntarily; he has had frequent attacks of retention of urine, and constant rigors. Since the application of the caustic, many ineffectual attempts have been made to introduce a bougie. An impermeable stricture was discovered about six inches down the urethra. At the request of Mr. C. Phillips, the author operated: he divided three fourths of an inch of the stricture with the single-lancetted stilette. The pain was trifling, and the bleeding amounted only to a few drops. On the next day, the patient had felt but little inconvenience, excepting, as had been customary with him after the use of a bougie, he was attacked by a slight rigor; he had made water in a small stream. The second day after the operation, a small-sized gum catheter passed easily into the bladder: it was left in the urethra. In ten days a No. 13 catheter passed without difficulty.

Case III.—The patient had laboured under stricture for two years. For a year, common bougies had been employed with advantage. Six months afterwards the stream of urine gradually diminished, until it was not larger than packthread. The stricture was six inches from the orifice, and was too tortuous to admit a metallic instrument. About half an inch of the stricture was divided with the single-lancetted stilette. The relief was immediate, and the stream of urine so much enlarged that the patient did not consider any further treatment necessary.

Case IV.—Thomas Facey, æt. forty-three, admitted into the Marylebone Infirmary, November 6th, 1828; has a hardened contraction of the orifice, and another, of about half an inch in length, an inch further on. Four inches from the orifice there is a permanent stricture, and also at the bulb; the urethra quite impermeable. Been strictured twenty years, and discharged as incurable from several hospitals. Is now much emaciated from the effects of the disease. Urine passes *guttatim*, constantly, and involuntarily.

Nov. 10th.—Mr. S. enlarged the orifice to its natural size, and divided the second stricture. A bougie was left in to prevent the parts from closing. Leeches to the perineum. In a few days the divided parts had healed, and the smallest bougie would pass through the fourth stricture, which before was impermeable.

On the 28th, Mr. S. operated over the wire, with the double stilette, upon the third stricture, about half an inch in extent; and also divided the fourth stricture at the bulb and membranous portion, more than an inch in length. Less than a tablespoonful of blood was lost, and the operation lasted two minutes. A small catheter left in the bladder. Rigor in the night, but no other unfavorable symptom. Urine had passed through and by the sides of the catheter.

December 1st.—No. 8 catheter introduced with ease into the bladder: in a few days, No. 11. In five weeks the cure was considered complete.

CASE V.—An impermeable stricture, about five inches down the canal, was divided with the single-lancetted stilette. Immediately after a catheter could be passed into the bladder. The man was a patient of Mr. VINCENT's, in St. Bartholomew's Hospital.

CASE VI.—John Edwards, æt. forty-nine, in the Mary-lebone Infirmary; strictured for some years; prepuce entirely adherent to the glans, and the orifice so closed and hardened that it would not admit a larger instrument than a No. 2 catheter. An impermeable stricture about five inches down the canal. Hardened edges of the prepuce were circumcised, and the prepuce itself dissected from the glans, and thrown back as in the operation for phymosis. A month elapsed before the parts healed sufficiently to allow of more being done. The orifice was then enlarged to its natural size, and kept open by the introduction of a bougie. A short time after the wire was introduced, and left in the urethra, and the operation was performed with the double-lancetted stilette. On the next day, the urine had been freely voided, but the urethra was too irritable to allow the passage of a catheter. At the end of three weeks, a No. 12 steel sound passed into the bladder with facility, and the patient was discharged cured.

CASE VII.—F. B., æt. sixty, a general officer; had been suffering from strictures for years, and lately they had greatly increased; prostate gland also enlarged and hardened. Other modes of treatment having failed, this gentleman determined to have the strictures divided with the lancetted stilette. In less than a fortnight from the operation, both the strictures would admit a steel sound, No. 15, into the bladder. Patient since continued well.

CASE VIII.—Much relieved by the operation, although the case appeared almost hopeless. The friends of the patient, being alarmed at so novel a plan, interfered, and prevented the cure from being completed.

CASE IX.—X. Y. Z. had an impermeable stricture five inches and three quarters from the orifice. Urine passed *guttatim*, sometimes dribbling involuntarily; he was obliged to rise once or twice every hour during the night to void it. Suffered several times from complete retention. Had undergone a course of bougies under ten surgeons of eminence, at different times, without avail. This patient was quickly relieved. He remained free from any unpleasant symptoms, and was able to retain his water for eight or ten hours.

Case X. presents nothing differing from those before mentioned. The treatment was successful.

Case XI.—Mr. C., a barrister, æt. thirty-seven, has had strictures for eighteen years, and for the last three years he has periodically gone through a course of bougies. Whenever he leaves off their use, however, the contraction returns. Profuse discharge from the urethra. A stricture behind the bulb would with difficulty admit the passage of the smallest sized gum elastic catheter through it. The author operated over the wire. The operation did not last a minute, and only a few drops of blood followed. The patient was astonished that he felt so little pain.

When the sheet containing the relation of this case was going to press, he was so rapidly improving that there could be no doubt of his speedy recovery.

To avoid repetition, we have not mentioned in each case one part of the treatment, which Mr. Stafford frequently finds necessary, both before and after the employment of the instruments. If the urethra is very irritable, and there are symptoms of local inflammation, with much general disturbance, local bleeding, opiate clysters, fomentations, &c. will be required.

The evidence that Mr. Stafford has now laid before the profession is certainly very strong in favor of the plan he proposes. We before felt ourselves called upon to speak with much caution upon the subject, and we still think that if ever these instruments are brought into general and common use, mischief will arise. We are confirmed in this belief when we bring to our recollection the many cases we have seen in which, from the *mal-adresse* of using even the common bougie, false passages in the urethra, and other injurious consequences, have been inflicted upon the patient. Of Mr. Stafford's skill and dexterity in the use of the instruments we have no doubt; and he has now so perfectly convinced us of their safety and efficacy, when they are dexterously employed, that, if we were ourselves labouring under an impermeable stricture, we should place ourselves under his care with the most perfect confidence.

*A Synopsis of Modern Medical Jurisprudence, anatomically, physiologically, and forensically, illustrated; for the Faculty of Medicine, Magistrates, Lawyers, Coroners, and Jurymen.* By J. S. Forsyth, Surgeon, &c. 12mo. pp. 600. Benning, London, 1829.

It is lamentable, but nevertheless it is very true, that the respectability and character of the profession have frequently suffered in the estimation of the public, from the very sorry part that medical practitioners have played when they have been placed in a court of justice, in the deeply responsible situation of witnesses in cases of medico-legal investigation. The vast importance of medical jurisprudence is universally acknowledged, but still it is more neglected by the great mass of practitioners than any other branch of medical science. It may be impossible, indeed, for the medical student, considering the variety of subjects that must necessarily engross his mind, to labour through many of the elaborate works that have been published upon this subject. Such a synopsis as the present he will therefore find a very useful addition to his library. The judge, the lawyer, and the juryman, will also find it a very convenient book of reference.

In the first chapter the author offers a few interesting remarks upon the leading points of the law of evidence. As medical men are so frequently called upon to give their testimony in a court of justice, they should at least be acquainted with the leading parts of the law of evidence, and particularly such as more immediately bear on their professional knowledge. It has been supposed that medical men may avail themselves of the privilege enjoyed by legal advisers, and that they are not bound to divulge the secrets of their patients, reposed in them in the course of professional confidence. Such confidence ought not to be violated on any ordinary occasion; but, when the ends of justice absolutely require the disclosure, there is no doubt that the medical witness is not only bound, but compellable to give evidence, ever bearing in mind that the examination should not be carried further than may be relevant to the point in question: of this the court will judge, and protect the witness accordingly. The manner in which a medical witness should give his evidence differs but little from that in which evidence ought to be given by any other profession or individual, the whole of which may be comprised in the following pertinent observations of Mr. Haslam,* whom

* Haslam on Insanity, London, 1817.

Mr. Forsyth quotes: "The important duty," he says, "which the medical practitioner has to perform when he delivers his testimony before a court of justice should be closely defined, conscientiously felt, and thoroughly understood; his opinion ought to be conveyed in a perspicuous manner; he should be solemnly impressed that he speaks upon oath, the most sacred pledge before God between man and man, and that the life of a human being depends upon the clearness and truth of his deposition; he is not to palm upon the court the trash of medical hypotheses, as the apology for crime; neither should the lunatic receive his cure at the gallows, by the infirmity of his evidence; but, above all, his opinion should be so thoroughly understood by himself, so founded by experience and fortified by reason, that it may resist the blandishments of eloquence, and the subtle underminings of a cross examination. The physician should not come into court merely to give his opinion; he should be able to explain it, and able to afford the reasons which influenced his decision: without such elucidation, his opinion becomes a mere dictum."

In the next chapter we enter into the immediate business of the volume, in which is discussed all the various subjects connected with forensic medicine. To give an analysis of a work which is itself, in a great measure, a condensed abridgment of other works, would be almost impossible. We merely wish to do fair justice to Mr. Forsyth's "Synopsis," by calling the attention of the profession to its general utility.

The fifth chapter contains some very good general rules for the examination of dead bodies, applicable to all cases where investigations are required. Before proceeding to dissection, it is proper first to examine the external situation and appearance of the body; and, to facilitate the investigation, the author lays down many important rules, which the surgeon will scrupulously attend to, if he consults his own character or the public interest.

"1. If death be apparently caused by a wound, the body should be first viewed, if possible, exactly in the position in which it was found. By moving it, the attitude of the extremities may be altered, or the state of a fracture or luxation changed, since the internal parts vary in their position with one another, according to the general position of the body. If it be absolutely necessary to remove the body, it should be done with great caution.

"2. The clothes should be removed, as far as is necessary, and it should be noted what compresses or bandages (if any) are applied to particular parts.

" 3. After these preliminaries, the colour of the skin should be examined; the temperature of the body, the rigidity or flexibility of the extremities, the state of the eyes and of the sphincter muscles; noting, at the same time, whatever swelling, ecchymosis,* wound, ulcer, contusion, fracture, or luxation, may be present; also any fluid flowing from the nose, mouth, ears, sexual organs, &c., and indeed every thing varying from the natural state. The above cavities should be inspected, and particular attention paid to the state of the skin, so as not to mistake that bluish brown tinge which indicates the commencement of putrefaction, for ecchymosis. This distinction will be presently explained.

" At this period it may sometimes be necessary to delay the dissection for a few hours; and the wish of the examiner is, of course, to prevent putrefaction. To effect this, it has been recommended to put the body in a cool place, and cover it with ice, or it may be sprinkled with spirituous or aromatic substances. If it be intended to preserve it for some time, it should be washed with a strong solution of common salt and alum. The cadaverous smell present during the examination may be obviated, in a great degree, if the change be not too far advanced, by passing a current of chlorine, or the fumes of tobacco, through the chamber; or what would appear, in these cases, to answer the purpose more effectively would be the chlorurets, as directed by M. Labarraque, and of which Mr. Alcock has recently given a very good account.

" From the period the dissector commences until he concludes, there should be some one at hand, to take down all the facts that from time to time may be communicated; and this should not be delayed until the examination be completed, as many circumstances of importance may otherwise escape the memory.

" *If there be an external lesion present,* it should first be examined, and its nature described, its length, breadth, depth; also whether it has been inflicted with a cutting, pointed, or round instrument; whether it be accompanied with inflammation or gangrene; and whether any foreign bodies are found in it, such as balls or pieces of cloth. The scalpel should then be employed to trace its extent, but with judgment, so as not to render our researches useless, and to prevent a comparison of the external wound with the internal injury. The nerves and blood-vessels, particularly the arteries that are wounded, should be named; as should also the viscera, if any are in that state.

" *If there be a contusion without a solution of continuity,* the injury found in the internal parts should be particularly noticed, such as extravasation, rupture of vessels, &c.

" *If the cause of death is a burn,* its degree and extent should be examined, together with the state of the parts affected, whether inflamed merely or covered with blisters; the fluid contained in

* " By ecchymosis is here meant a black and blue swelling, either from a bruise or spontaneous extravasation of blood.

these blisters, and the condition of the neighbouring parts, whether sphacelated or gangrenous.

" *If a luxation or fracture* is present, notice the surrounding soft parts, the nature of the injury, whether simple or complicated, and the phenomena indicating the progress of disease or of recovery.

" Having stated all these circumstances, it is next necessary to proceed to the dissection in a systematic manner; and the rule here is to commence with an examination of the abdomen, as that part is most liable to run into putrefaction. We, however, should not desist because the cause of death is supposed to be perfectly discovered in one or the other cavity: all of them should be investigated.

" *Inspection of the head.*—On shaving the head, the integuments are to be inspected, and all injuries done to them are to be noticed. In particular, if a wound appear to be inflicted by a sharp-pointed instrument, its depth, direction, and connexion with the brain, should be minutely traced. The presence of inflammation, œdema, or sphacelus, must also be remarked. The same observations apply to injuries from cutting instruments. The bones of the cranium are next to be laid bare; search is to be made for fissures or fractures, taking care, at the same time, not to mistake irregular sutures for them; and for this purpose they should be rubbed over with ink. The strength of these bones is also deserving of minute inspection, as they not unfrequently are so thin or soft as to render a blow very destructive, but which, under ordinary circumstances, would only produce slight injury. The fracture should always be followed throughout its whole extent.

*The brain and the membranes* must be carefully inspected. Let it be noticed whether any matter or blood be interposed between the dura mater and the bones, or whether it is detached or inflamed. All morbid appearances in structure deserve attention, and the state of the vessels of the brain, the fluids present, and their situation, together with the changes in the substance, are highly deserving of attention. It should, however, be remembered that death sometimes follows from blows on the head, when no internal lesion can be found on dissection. It has been abundantly proved that the connexion between the brain, the viscera of the thorax and abdomen, are the cause of this, and the injury therefore is to be looked for in the latter.

" *The vertebral column* must be viewed throughout its whole extent, to ascertain whether it be fractured, dislocated, or contused. This part requires strict attention, since injuries of it are often of a very complicated nature.*

* " Foderé quotes a case from Jaegar, of a person who was struck on the neck by a loaded waggon, with such violence that both his upper and lower extremities became paralytic. He died in eighteen hours after the accident. No external appearances of injury could be observed, although an examination readily indicated that the seat of disease was somewhere near the sixth

" *The neck*.—We should carefully inquire whether the neck bears any marks of external injury, or traces of ecchymosis or pressure on it. Examine the great blood-vessels, whether they are filled with blood or empty; and the nerves, whether they are in their natural state. The œsophagus, pharynx, and larynx, must be examined. If wounded, detail the extent, depth, and shape of the injury, and particularly if the lesion is caused by fire-arms; its course, and the loss of substance present, with the inflammation or suppuration (if any) existing, should be stated.

" *The thorax*.—On proceeding to the thorax or chest, it should first be ascertained whether the injuries it has received are superficial, affecting the integuments and muscles merely, or whether they extend to its cavity. If the latter, the following are subjects worthy of observation: The blood-vessels, the bones covering the thoracic viscera; inquire whether any of these are fractured, luxated, or diseased. Next, as to the respective organs, notice the lungs, and their internal as well as external condition; the pericardium and its contents; the heart and its great vessels; the diaphragm, the thoracic duct, and the phrenic nerves: all injuries or diseases of these should be stated in the report.

" *The abdominal cavity*.—The external covering of the abdominal cavity forms a subject of inquiry. Every spot, swelling, or extravasation, should be noticed, as also whether hernia be present, or whether there is any tumefaction of the part. The organs peculiar to either sex should be examined, and also the various viscera contained in this cavity: the stomach, mesentery, liver,* spleen,† pancreas,‡ kidneys,§ gall-bladder, intestines, bladder, &c. Each part should be viewed as to the quantity of blood contained in it, and which naturally belongs to it; all extravasations should be traced as to their quantity and nature; and particular inquiry should be made whether the changes observed are the cervical vertebra; and accordingly, on dissection, its spinous apophysis was found broken at its base, and separated from its body, while blood was extravasated to the amount of four ounces.

* " The liver may present several morbid phenomena, which, in a dissection instituted for the purpose of discovering the cause of death, ought not to be overlooked. It may also be found ruptured; an occurrence which may take place where little or no external injury can be perceived, as from a sudden fall, or from the application of strong pressure applied to the upper part of the abdomen, such as might be occasioned by the passage of a heavy carriage over the body. See Med. Trans. of the College of Physicians, for an interesting case of this kind, vol. iii. p. 577, by Dr. G. Pearson.

† " The spleen may be brought into view by drawing the stomach towards the right side, when the one viscus will follow the other. This organ, like the liver, may be ruptured by violence.

‡ " The pancreas is to be seen by tearing through the great omentum, between the large curve of the stomach and the arch of the colon.

§ " The appearance of the kidneys, although not generally an object of dissection, ought to be noticed, as it is frequently connected with the exhibition of poisons. Like the other solid viscera, too, the kidneys may be ruptured by external violence, and several instances are on record to this effect.

result of disease or of sudden injury. In examining the intestines, Professor Mahon recommends the use of a blunt-pointed bistoury, as this may prevent injury during the dissection. The contents of the stomach and intestines should be analyzed, and those of the gall-bladder and urinary bladder inspected." (P. 73.)

In females, the organs of generation should always be inspected, as very important conclusions may be deduced from the discovery of an unimpregnated uterus. In death from hemorrhage, or from any other cause in childbirth, the appearances that will present themselves are well described by Professor BURNS. Those parts from whose condition or appearance any legitimate deduction can be made, should be preserved. In cases of poisoning, the stomach and intestines, with their contents, should be kept for subsequent experiments.

It will be perceived how essentially necessary an accurate knowledge of anatomy is in such cases, that the appearances which are natural may not be mistaken for extraordinary occurrences, or the effects of disease for those of violence. We would particularly enforce the necessity of perusing with attention the paper of Dr. EBERMAIER upon perforations in the stomach arising from obscure disease.* The appearances that are found upon dissection in such cases, taken in connexion with the sudden occurrence and rapid fatality of the symptoms, have more than once led to the suspicion that poison had been administered. A case of this nature lately happened in France, in which the life of an innocent person was placed in the utmost jeopardy, from the ignorance of the medical witnesses upon this very important subject.

* Vide London Med. and Phys. Journal for October 1828, p. 302; and the succeeding Number, p. 422.

